# Prevalence of Anemia Among Children and Adolescents in Rural Area of Khulais in Saudi Arabia

**DOI:** 10.7759/cureus.21894

**Published:** 2022-02-04

**Authors:** Badr M Madani, Abdulslam M Alsulami, Ibrahim A Abu alola, Saad M Alasmari, Faisal M Khoujah, Osama M Andijani, Mshari A Alhomrani, Sari S Alhomrani, Saeed S Alquhaibi, Faisal S Alsulami, Waleed A Ibrahim, Bakor A Abu alola, Ali H Alqarni

**Affiliations:** 1 Family Medicine, University of Jeddah, Jeddah, SAU; 2 Medicine, University of Jeddah, Jeddah, SAU; 3 Medicine and Surgery, University of Jeddah, Jeddah, SAU

**Keywords:** saudi, adolescents, children, rural area, anemia

## Abstract

Introduction

The decreased absolute circulating red blood cell count or the inability of red blood cells to meet physiological needs is called anemia. Anemia can affect mental health, learning capacity, and the ability to concentrate. The present study aimed to assess the prevalence of anemia among children and adolescents living in the rural areas of Khulais, Saudi Arabia.

Methods

This cross-sectional study including 417 individuals was conducted at Khulais Hospital in the rural areas in Saudi Arabia to estimate the prevalence of anemia among children and adolescents. The inclusion criterion for the study was that participants must be Saudi citizens. Data for this study were collected in March 2021. The age of the children ranged from 7 to 11 years, whereas that of adolescent males and females was between 12 and 18 years.

Results

In total, the study included 147 male adolescents, 123 female adolescents, and 147 children participants. The overall prevalence of anemia among adolescents was high (39.1%). The prevalence of anemia was 44.9% (66/147), 46.3% (57/123), and 27.2% (40/147) in male adolescents (age 12-18 years), female adolescents (age 12-18 years), and children (age 7-11 years), respectively. Statistical analysis revealed an association between the prevalence of anemia and the increasing age of participants from rural areas.

Conclusion

The present study results indicate that the prevalence of anemia in the rural areas of Saudi Arabia is high. The high prevalence can be explained by several factors, such as parents’ low socioeconomic status and living in rural areas, which limits the availability as well as different types of nutritious food and thereby negatively affects the nutritional status of such children and adolescents.

## Introduction

Anemia is defined as a condition in which the absolute number of circulating red blood cells is reduced or the red blood cells along with their oxygen-carrying capacity are insufficient to meet physiological needs [1]. While anemia is typically diagnosed as low hemoglobin (Hb) or low hematocrit concentrations [2], it can also be diagnosed based on changes in blood reticulocyte count, mean corpuscular volume, blood film analysis, or Hb electrophoresis [3]. Nonetheless, given the population levels and constraints of clinical practice, Hb concentration is the most commonly used hematological assessment to define anemia [4]. Anemia has a high prevalence globally and can develop at all stages of life, particularly in preschool-aged children [5]. According to the World Health Organization (WHO) cutoffs, anemia is defined as a Hb concentration of <11 g/dL in girls and <12 g/dL in boys [6]. Of note, anemia can affect mental health, learning capacity, and the ability to concentrate [6].

Iron deficiency is the most common cause of anemia, and available evidence from clinical and epidemiological research demonstrates that iron deficiency anemia (IDA) is significantly associated with a higher risk of unipolar depressive disorder, bipolar disease, anxiety disorder, attention deficit-hyperactivity disorder, delayed development, and mental retardation among both children and adults [7].

The highest global prevalence of anemia is found in Africa (47.5%), followed by South-East Asia (35.7%) [8]. Moreover, its prevalence is approximately 17.8% in the Americas, 14% in the United Arab Emirates, 11% in Egypt, and >40% in the Syrian Arab Republic and Oman among women of childbearing age [8]. Several studies from Saudi Arabia on anemia have been based on nutritional status in preschool children aged <6 years [8]. This study was conducted to determine the prevalence of anemia in Saudi Arabia based on age and the type and severity of anemia.

## Materials and methods

This retrospective cross-sectional study including 417 individuals was conducted at Khulais hospital, a tertiary government hospital that provides free healthcare facilities to Saudi citizens and residents in Saudi Arabia, to estimate the prevalence of anemia among children and adolescents.

The inclusion criterion for the study was that participants must be Saudi citizens. Data for this study were collected in March 2021. Information on sex, age, Hb concentration, and mean corpuscular volume was obtained from patient records in the hospital. Participants were classified into three groups: children, male adolescents, and female adolescents. The age of the children ranged from 7 to 11 years, whereas that of the adolescent males and females was between 12 and 18 years. Ethical approval for this study was obtained from the Bioethics Committee of Scientific and Medical Research, the University of Jeddah (approval number, U-REc-0os; approval date, 14/9/2021). 

Data collection

Hemoglobin levels, age, sex, and mean corpuscular volume (MCV) were obtained from hospital archive files. According to WHO guidelines, anemia was defined as a hemoglobin concentration below 11 g/dL, and it was classified as mild (10.0-10.9 g/dL), moderate (7.0-9.9 g/dL), or severe (<7.0 g/dL). Microcytic anemia is defined as MCV less than 80 femtoliters (fl), normocytic anemia as MCV from 80-100 fl, and macrocytic anemia as MCV more than 10 0 fl.

Statistical analysis

The SPSS statistical software suite, IBM SPSS statistics version 19 (IBM Corp., Armonk, NY) was used to analyze the data. Based on the WHO criterion of 12 g/dL, the outcome variable was classified as anemic or nonanemic. The mean, standard deviation, and proportions were calculated as descriptive statistics. The number of children, age, and Hb level were treated as continuous variables.

## Results

**Figure 1:**
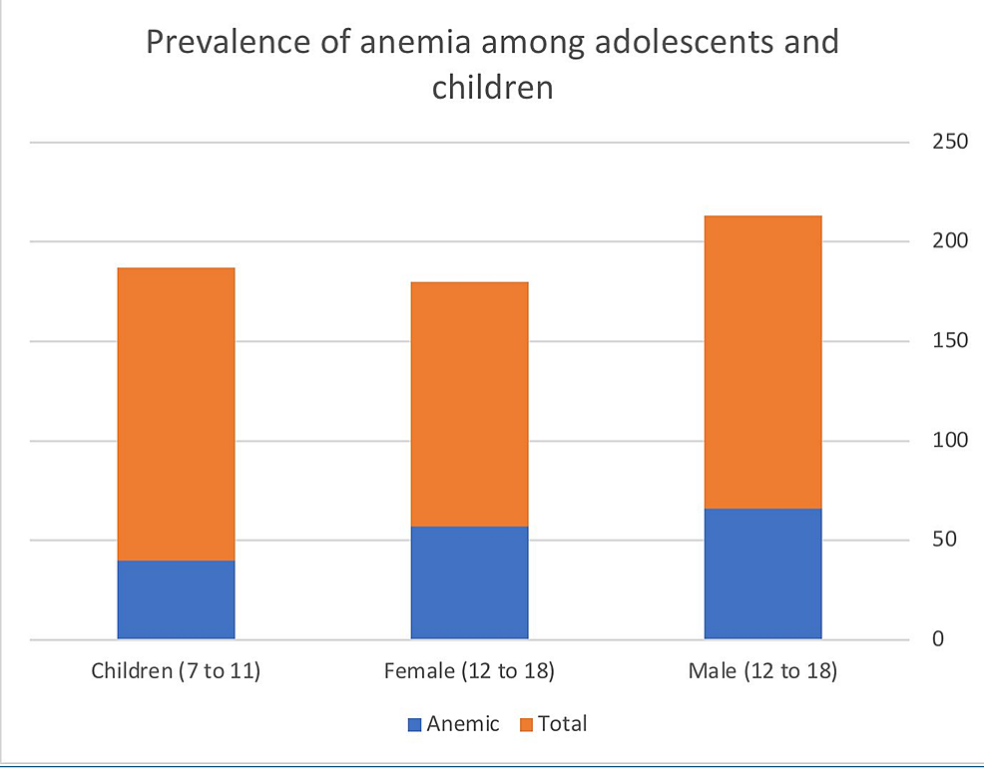
Prevalence of anemia among adolescents and children

This study included the data of 417 individuals. Their median age was 14 years, with a standard deviation of 3.77, and the median value of Hb concentration was 12.7 g/dL. The study population comprised 147 adolescent males, 123 adolescent females, and 147 children. The overall prevalence of anemia among adolescents was high: the prevalence among male adolescents was 44.9% (66/147), among female adolescents was 46.3% (57/123), and among children was 27.2% (40/147) (Figure 1). 

**Figure 2:**
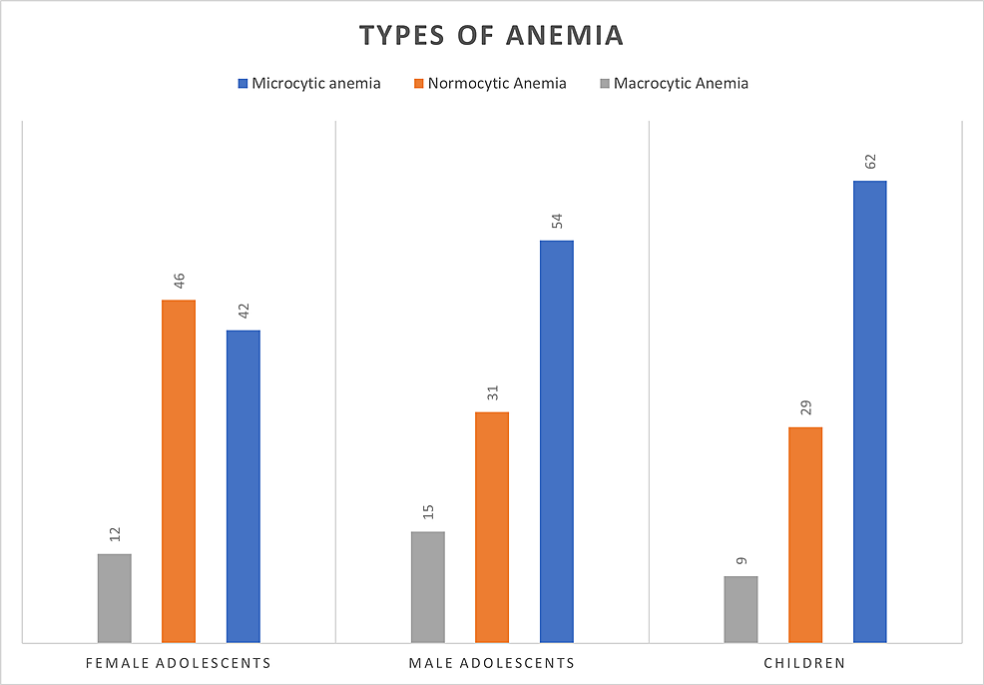
Types of Anemia

These results indicate a potential association between the prevalence of anemia and the increasing age of participants from rural areas. Anemia among children was predominantly microcytic (62%), followed by normocytic (29%); only 9% of the children showed macrocytic anemia. Additionally, while the most common type of anemia among male adolescents was microcytic anemia (54%), approximately 46% of adolescent females had normocytic anemia (Figure 2).

Although none of the children had severe anemia, approximately 25% had moderate anemia (Hb = 8-10 g/dL) and 75% had mild anemia (Hb > 10 g/dL). In contrast, although severe anemia (Hb < 8 g/dL) was observed in 8.77% of female adolescents, 16% had moderate anemia and 75.4% had mild anemia. Furthermore, 1.5% of male adolescents had severe anemia, 9.09% had moderate anemia, and 89.4% had mild anemia.

## Discussion

The present study aimed to estimate the prevalence of anemia among children and adolescents in rural areas in Saudi Arabia, and the findings revealed that the prevalence of anemia increased with increasing age (up to 18 years) in both males and females. These findings are in agreement with results from WHO, which state that anemia incidence rates were higher in older men than in older women and that they increased with increasing age [9]. Further, nearly 80% of the patients with anemia subsequently had lower Hb concentrations during follow-up [10]. We showed that the prevalence of anemia was higher among female adolescents than among male adolescents, which is in agreement with the results reported by the National Heart, Lung, and Blood Institute and the Centers for Disease Control and Prevention. These observations are attributed to menstrual blood loss; in particular, while a typical menstrual bleeding phase continues for 4-5 days and the amount of blood lost ranges from 2 to 3 tablespoons, females with excessive menstrual bleeding can bleed for >7 days and lose twice the normal amount of blood, which is an established risk factor for developing anemia [11]. 

Furthermore, approximately 20% of women of childbearing age suffer from IDA, and pregnant women are even more likely to develop IDA owing to the greater demand for nutrients because of the growing fetus. Of note, the prevalence of anemia among males in our cohort was lower than that reported by others [11]. In males, the attainment of adult testosterone concentration is linked to an increase in erythropoiesis [10]; thus, the lower prevalence of anemia among adolescent males can be explained by a physiological increase in Hb concentration secondary to sexual maturation as well as lower Hb demand once the growth spurt has ended [11-12].

The most common causes of anemia among different age groups include suboptimal diet and lifestyle, and multiple studies in various countries have provided evidence to support this finding. For example, a cross-sectional study among school-going adolescents in Bonga Town, Southwest Ethiopia, revealed that the prevalence of anemia was 15.4% among children who consumed meat less than twice a week and 12.8% among those who ate meat twice or more every week [13]. Another study on adolescents aged 12-19 years in Denizli, Turkey, reported an anemia prevalence of 87% among children eating meat once a week but only 13% among those consuming meat twice or more per week [14]. Another cross-sectional study among adolescent girls aged 10-19 years and living in an urban area in Mumbai, India, showed that anemia was prevalent in 91.5% of the participants who consumed fruits once a week and 71.6% among those who ate fruits twice or more per week. Likewise, anemia prevalence was 90.9% among those consuming green leafy vegetables once a week and 74.8% among those eating green leafy vegetables twice or more per week [15].

The most common type of anemia among the children in our study was microcytic anemia, and iron deficiency was its most common cause. IDA is a widespread and common nutritional disorder worldwide that has persisted despite all efforts to reduce its prevalence. For example, it has been estimated that the prevalence of IDA is 53.6% in Iran, 60% in Africa, 46% in Latin America (46%), 63% in the Eastern Mediterranean region (63%), and 49 and 66%, respectively, in Southeast Asia I and Southeast Asia II. Moreover, a high prevalence of IDA has been reported in Tanzania (79.6%), Kenya (35.3%), and Nigeria (82.6%) [16-19].

## Conclusions

The present study results indicate that the prevalence of anemia in the rural areas of Saudi Arabia is high and that it is more prevalent among female adolescents than among male adolescents. Furthermore, there appear to be gender-specific differences in the type of anemia. Certain factors, including young age, positive family history of IDA, lack of healthy diet intake, and low socioeconomic status, are probably associated with the prevalence of anemia, and it may be challenging to address all these factors together. Thus, corrective measures to reduce the prevalence of anemia should focus on key factors such as the family history of IDA and low socioeconomic status. Finally, early identification and family screening are important approaches that can reduce the burden of this disease, and the reduction can be accomplished via various methods, including annual mass screening programs and school health programs with subsequent outreach to other family members.
